# Accuracy of face scan‐based virtual facebow records: A comparison between two registration methods

**DOI:** 10.1111/jopr.70153

**Published:** 2026-05-06

**Authors:** Mario Hertanto, Marcel Hertanto, Zhiqiang Luo, Fei Liu, Luca Lepidi, Junying Li

**Affiliations:** ^1^ Department of Biologic & Materials Sciences and Prosthodontics University of Michigan School of Dentistry Ann Arbor Michigan USA; ^2^ Department of Prosthodontics University of Ferrara Ferrara Italy

**Keywords:** in vitro, iterative closest point algorithm, landmark‐based registration, precision, surface‐based registration, three‐dimensional facial imaging, trueness, virtual articulator mounting

## Abstract

**Purpose:**

Accurate maxillary positioning is critical in digital prosthodontics, particularly when transferring the spatial relationship of the maxilla to a virtual articulator. This study aimed to compare the accuracy of the two most common registration methods, three‐dimensional (3D) match and UV (landmark‐based point‐pair registration) match, for virtual facebow transfer and to investigate whether the type of facial scanner used influences the accuracy of these registration techniques.

**Materials and Methods:**

A cone beam computed tomography (CBCT) scan of a manikin was used as the reference. The maxillary arch was scanned with three anatomical landmarks and aligned to the CBCT. A computer‐aided design and computer‐aided manufacturing (CAD‐CAM)‐printed facebow fork was affixed to the arch, followed by facial scans using the EinScan HX and iPad (10 scans each). For each scan, two registration methods (3D match and UV match) were performed, generating 40 virtual alignments. Superimpositions were completed in Exocad, and accuracy was evaluated by comparing each registration to the CBCT reference using a Python script to measure linear and angular deviations. Trueness and precision were analyzed using a linear mixed model.

**Results:**

3D match generally outperformed UV in both trueness and precision. The industrial scanner combined with 3D match yielded the highest accuracy, with linear trueness of 0.98 ± 0.67 mm and precision of 1.31 ± 0.79 mm (*p* < 0.001). With the iPad, 3D registration demonstrated significantly superior angular accuracy (*p* < 0.050).

**Conclusion:**

3D registration provides higher accuracy than UV match, and 3D registration, in combination with an industrial‐grade scanner, offers higher accuracy in virtual facebow transfer than using iPad.

The precise transfer of the maxillary arch position is central to prosthodontic diagnosis, treatment planning, and execution. Conventional mechanical facebows can present limitations, particularly in patients with skeletal asymmetry, because articulators are constructed with symmetrical posterior references; when posterior landmarks are not level, the transfer and mounting process may introduce occlusal plane canting and midline deviation.^1^ In contrast, digital facebow records acquired with 3D facial scanning have demonstrated clinically acceptable trueness and precision for transferring the maxillary arch to a virtual articulator, potentially reducing inaccuracies associated with analog registration and cast‐mounting steps.^2^ More broadly, integrating digital tools into prosthetic workflows can streamline clinic–laboratory data transfer and improve efficiency and predictability.^3^ This is particularly significant in extensive prosthetic rehabilitations involving esthetic considerations, where accurate 3D spatial positioning of the maxillary arch is crucial.^1^ In fact, maxillary cast alignment is the most crucial step in the digital workflow, where its accuracy impacts all following steps, including virtual articulator mounting, esthetic profile design, and occlusal plane position.^2^

The integration of various digital technologies has driven the development of 3D virtual patients, which is beneficial for facially driven restorative treatment planning.^4^, ^5^ This trend is increasingly employed for diagnosis and treatment planning,^2^ and its clinical procedure employs a facebow fork, which includes extra‐oral and intraoral reference markers. A virtual patient is created by aligning the patient wearing a facebow fork with the scan of a facebow fork alone.^2^ The benefit of a facially driven and prosthetic treatment plan leads to prosthetic and successful treatment longevity. However, the accuracy of the virtual patient workflow must be critically evaluated to identify any possible distortion at each step, starting from data acquisition and virtual alignment to manufacturing.^6^

Li et al. emphasize the importance of integrating occlusion and virtual articulator systems within the digital workflow to ensure accurate registration of the maxillomandibular relationship. Their study also highlights the need for precise alignment of facial and intraoral scans. Therefore, the significance of accurate data acquisition and integration cannot be overstated, as any errors may compromise the treatment outcomes.^7^

There are two commonly employed registration methods: 3D match (surface‐based, best fit) and UV match (landmark‐based or point‐pair registration).^8^ In this study, UV match refers to manual selection of more than three corresponding landmarks or points on the two meshes to compute the final rigid transformation. The best fit alignment uses the iterative closest point (ICP) algorithm, which registers two 3D surfaces by iteratively computing a transformation matrix that coordinates the first surface to the closest points of the second surface, and reduces reliance on landmarks to achieve the correct final alignment,^9^ whereas the matching process of point‐related or UV matching is based on corresponding landmarks.^8^ UV match or point‐based registration is a direct and semi‐automatic method based on the selection of at least three corresponding landmarks on two 3D objects. Subsequently, a mathematical algorithm transfers the 3D objects to find the best fit for point pairs.^10^

Many studies have supported the accuracy of face scans and virtual facebow. Inoue et al. found that 3D facial scans perform similarly to traditional facebow in virtual mounting dental casts.^11^ Recent clinical research by Al Yousuf et al. found that virtual facebow records made with a smartphone are more accurate than a conventional facebow.^12^ However, the influence of different registration methods on the accuracy of virtual facebow transfer remains unclear. Therefore, this study aims to compare the accuracy of two registration methods, 3D match and UV match, in face scan‐based virtual facebow records, and to investigate if there is an association between the facial scanning device used and registration method in the accuracy. The null hypothesis is that there is no difference in accuracy between registration methods and no association between facial scanning devices and registration method used.

## MATERIALS AND METHODS

This prospective in vitro study was conducted using a manikin head, which was assembled using a rubber facial skin shroud (Replacement Skin Rubber Face for Dental Manikin, item #CDXPH‐2; Columbia Dentoform) fitted over a life‐size skull model (360° Rotatable Life Size Human Skull on Cervical Vertebrae Anatomical Model, SKU M0048; Medarchitect). The printed facebow fork used in this study was adapted from previous research^2^ and fabricated using a 3D printer (Form 3+ 3D printer; Formlabs) with model resin (Model Resin V2; Formlabs). A post hoc power analysis was performed based on the effect size estimates from previous studies^2^, ^12^ and pilot data to evaluate the power to detect significant differences between registration methods. Given the repeated‐measures design with two within‐subjects factors (scanner type and registration method), G*Power 3.1 (Heinrich‐Heine‐Universität Düsseldorf) software was used to compute the achieved power for the observed effect sizes. For the primary outcome of linear trueness, the effect size (Cohen's *f*) was calculated based on the observed mean differences and pooled standard deviations between the 3D match and UV match groups. With *α* = 0.05 and 10 repeated scans per device, the analysis indicated that the study achieved 80% power to detect a medium‐to‐large effect size (*f* ≥ 0.40) for the main effects of registration method and scanner type. The observed effect sizes for linear trueness (iPad: *f* = 0.18; industrial scanner: *f* = 0.45) and angular trueness (iPad: *f* = 0.68) exceeded the detectable threshold, confirming that the study was adequately powered for the primary comparisons of interest.

**FIGURE 1:**
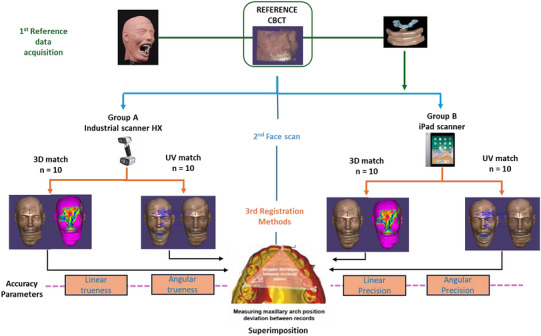
Research design flow chart.

Scanning using an HX industrial scanner (EinScan HX 3D scanner; Shining 3D) was done 10 times, and scanning with an iPad (iPad; Apple Inc) was done 10 times. An iPad with a 3D scan application (Heges 3D Scanner app; Marek Šimoník) was utilized to scan the face (Figure 1).

**FIGURE 2:**
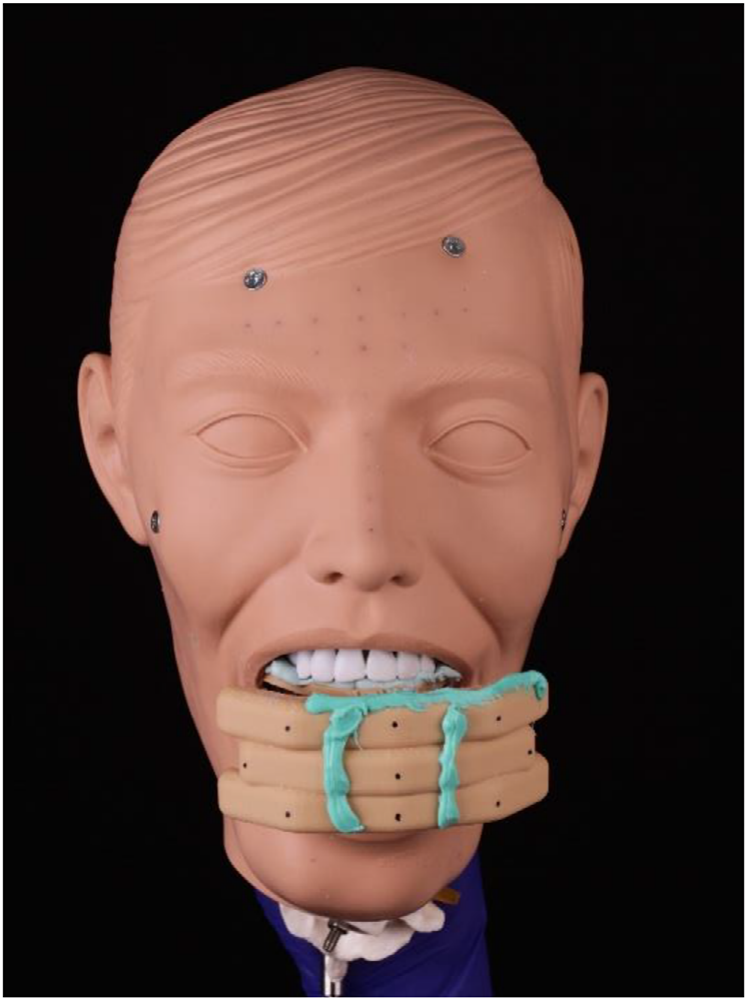
Manikin biting a 3D‐printed facebow fork.

Facial scans were repeated 10 times, with the manikin biting a 3D‐printed facebow fork, which was secured into the maxillary arch using a polyvinyl siloxane occlusal registration material (Futar; Kettenbach LP) (Figure 2). This virtual facebow technique was adapted from Solaberietta et al.,^13^ and the facial scan using intraoral scan bodies was adapted from Pérez‐Giugovaz et al.^4^ Finally, the facebow fork was removed and scanned using an intraoral scanner.

A scan mesh density of 1 mm was used as a standard for facial scanning, and the operator placed the iPad approximately 30 cm from the manikin's face. The manikin's face contained dots centered around the glabella because the glabella appears to be a soft tissue landmark that is well captured by facial scans and provides a reliable indication, as seen by consistency in the Inoue et al. study.^11^ The scan strategy began at the midline of the face, moved to the left side until the tragus was captured, and then proceeded to the right side until the tragus was captured. Then, the scan was repositioned back to the center of the midline and moved up and down, passing the facial midline until trichion and menton were obtained. The 3D model was exported as a standard tessellation language (STL) file.

An intraoral scanner (TRIOS 3 with Color Pod; 3Shape) was used to scan the manikin's maxillary arch using 3Shape Unite software (v23.1.0; 3Shape), and the files were exported in STL format. The scans were imported into an open‐source 3D computer software (Blender, version 2.80; The Blender Foundation). Three reference points with a spherical diameter of 0.5 mm were placed on the mesiobuccal cusp of the left maxillary molar (tooth #14), the mesiobuccal cusp of the right maxillary molar (tooth #3), and the mesioincisal angle of the left maxillary central incisor (tooth #9) for future reference to measure the discrepancy between experiment and reference. This measurement approach was adopted from previous research.^2^

All four scans—face scan, face scan with bite fork, printed bite fork, and intraoral scan (IOS)—were superimposed to obtain the position of maxillary dentition using Exocad software (exocad DentalCAD software; exocad GmbH). Two registration protocols were evaluated: surface‐based best‐fit registration (3D match) and landmark‐based point‐pair registration (UV match).

For the 3D match, datasets were first approximated using point‐to‐point alignment, then the UV alignment mode provided by Exocad was chosen, which performs alignment. Following that, the refinement was conducted using the 3D registration function available in Exocad, which is the best fit. This same workflow was applied consistently for all superimpositions.

For UV match, the superimposition of the manikin facial scan with the manikin biting printed bite fork and the superimposition of the manikin biting‐printed facebow fork with the printed bite fork were performed using landmark‐based point‐pair alignment by selecting more than six corresponding landmarks on each dataset. The alignment was accepted after visual verification of congruence in the regions of interest.

All alignments were performed by a single operator, who routinely performs alignment on a daily basis. A standardized protocol was followed^4^ and the alignment was performed consistently at the same time, using the same device to eliminate interoperator variability and minimize intraoperator variability. The alignment of manikin to manikin biting facebow fork used black marks of the forehead and nose as a standard reference.^6^

There were four experimental groups: an industrial scanner with best‐fit matching (HX_3D match), an industrial scanner with UV match (HX_UV match), an iPad with best‐fit matching (IP_3D match), and an iPad with UV match (IP_UV match).

The location of the maxillary dentition was compared to the reference of cone beam computed tomography (CBCT) as a baseline. The measurement and calculation were performed automatically using a Python script (Python Software Foundation) operated by J.L. Thus, human errors during calculation were removed.

Statistical analysis was performed using software (IBM SPSS Statistics v.30; IBM Corp.). Descriptive statistics were carried out with means, medians, and standard deviations. Linear deviations (in millimeters) were measured at three predefined reference points (#3, #9, #14), within the same scan. Due to the repeated‐measure nature of the linear deviations (three teeth per scan), a linear mixed model was used. Angular deviation between the occlusal planes in degrees was measured once per scan (one occlusal plane angle relative to the CBCT reference). Since there are no repeated measures within a record, the group differences were assessed using a linear regression. Each model was used to test for differences using a significance level of *α* = 0.05. Accuracy was assessed on four parameters: linear trueness, angular trueness, linear precision, and angular precision.

## RESULTS

A total of 20 scans and 40 alignment attempts were completed. The definition of accuracy is set according to the International Organization for Standardization (ISO 5725‐1), which states that accuracy involves trueness and precision, where trueness represents how close the measurement is to reality, and precision is reproducibility between measurements.^14^ The accuracy parameter was set based on four criteria: linear trueness, angular trueness, linear precision, and angular precision.

Table 1 presents the trueness of virtual facebow records obtained using an iPad scanner, comparing 3D registration (IP_3D match) with UV registration (IP_UV match). The average linear deviation at three reference points for the IP_3D match was 1.48 ± 0.86 mm, while the IP_UV match was 1.60 ± 0.90 mm. No statistically significant linear difference was found between the two groups (*p* = 0.349), and the difference was statistically significant (*p* = 0.002).

**TABLE 1:** Trueness of virtual facebow records by iPad scanners: 3D match (surface‐based) versus UV match (landmark‐based).

	3D registration			UV registration			
	Mean ± SD	Median	Range	Mean ± SD	Median	Range	*p*
3D linear deviation (mm)							
Tooth #3	1.60 ± 1.02	1.69	3.00	1.67 ± 1.18	1.25	3.31	
Tooth #9	1.56 ± 0.93	1.61	2.83	1.54 ± 0.81	1.31	2.30	
Tooth #14	1.26 ± 0.60	1.23	1.75	1.58 ± 0.75	1.40	2.58	
Combined	1.48 ± 0.86	1.48	3.04	1.60 ± 0.90	1.33	3.31	0.349^a^
Angular deviation (degree)	1.03 ± 0.51	1.13	1.53	1.88 ± 0.67	2.02	1.94	0.002^b^

^a^ Linear mixed model comparing the difference between two groups for linear deviation: *p* < 0.05.

^b^ Linear regression comparing the difference between two groups for angular deviation: *p* < 0.05.

Table 2 presents the precision of virtual facebow records obtained using an iPad scanner, comparing 3D registration (IP_3D match) with UV registration (IP_UV match). The average linear deviation between the three reference points for the IP_3D match was 2.09 ± 0.93 mm, while the IP_UV match was 1.99 ± 1.36 mm. However, the mean difference was not statistically significant (*p* = 0.509). Regarding angular deviation, the IP_3D match was superior to the IP_UV match, and the difference was statistically significant (*p* = 0.025).

**TABLE 2:** Trueness of virtual facebow records by industrial scanners: 3D match (surface‐based) versus UV match (landmark‐based).

	3D registration			UV registration			
	Mean ± SD	Median	Range	Mean ± SD	Median	Range	*p*
3D linear deviation (mm)							
Tooth #3	1.09 ± 0.87	0.82	2.76	2.17 ± 2.54	1.13	8.25	
Tooth #9	0.93 ± 0.68	0.87	2.10	1.52 ± 1.00	1.41	2.61	
Tooth #14	0.92 ± 0.46	0.86	1.22	1.99 ± 1.94	1.30	6.49	
Combined	0.98 ± 0.67	0.84	2.83	1.48 ± 1.07	1.01	3.58	<0.001^a^
Angular deviation (degree)	1.06 ± 0.40	1.03	1.24	1.36 ± 0.61	1.14	1.62	0.248^b^

^a^ Linear mixed model comparing the difference between two groups for linear deviation: *p* < 0.05.

^b^ Linear regression comparing the difference between two groups for angular deviation: *p* < 0.05.

Table 3 presents the trueness of virtual facebow records by industrial scanners between 3D registration (HX_3D match) and UV registration (HX_UV match). The average linear deviation at three reference points for the HX_3D match was 0.98 ± 0.67 mm, whereas the HX_UV match was 1.48 ± 1.07 mm. The difference between these two registration methods was statistically significant (*p* < 0.001). Regarding angular deviation, HX_3D shows better than HX_UV, but the difference was not statistically significant (*p* = 0.248).

**TABLE 3:** Precision of virtual facebow records by iPad scanners: 3D match (surface‐based) versus UV match (landmark‐based).

	3D registration			UV registration			
	Mean ± SD	Median	Range	Mean ± SD	Median	Range	*p*
3D linear deviation (mm)							
Tooth #3	2.38 ± 1.05	2.45	4.04	2.37 ± 1.48	2.17	6.77	
Tooth #9	2.21 ± 0.90	2.12	3.57	1.80 ± 1.21	1.25	5.44	
Tooth #14	1.68 ± 0.68	1.63	2.73	1.81 ± 1.33	1.41	4.91	
Combined	2.09 ± 0.93	1.99	4.04	1.99 ± 1.36	1.49	6.83	0.509^a^
Angular deviation (degree)	1.24 ± 0.60	1.23	2.39	1.59 ± 1.21	1.23	4.69	0.025^b^

^a^ Linear mixed model comparing the difference between two groups for linear deviation: *p* < 0.05.

^b^ Linear regression comparing the difference between two groups for angular deviation: *p* < 0.05.

Table 4 indicates the precision of virtual facebow records by industrial scanners between 3D registration (HX_3D match) and UV registration (HX_UV match). The average linear deviation between the three reference points for the HX_3D match was 1.31 ± 0.79 mm, while the HX_UV match was 2.60 ± 2.16 mm. Based on this outcome, the HX_3D match was superior to the HX_UV match, and the difference was statistically significant (*p* < 0.001). However, there was no significant difference between the HX_3D match and the HX_UV match regarding angular deviation (*p* = 0.076).

**TABLE 4:** Precision of virtual facebow records by industrial scanners: 3D match (surface‐based) versus UV match (landmark‐based).

	3D registration			UV registration			
	Mean ± SD	Median	Range	Mean ± SD	Median	Range	*p*
3D linear deviation (mm)							
Tooth #3	1.52 ± 0.95	1.54	3.43	3.03 ± 2.64	2.06	8.71	
Tooth #9	1.34 ± 0.79	1.45	2.98	2.09 ± 1.15	1.91	4.07	
Tooth #14	1.07 ± 0.53	1.15	1.83	2.69 ± 2.33	1.90	8.22	
Combined	1.31 ± 0.79	1.32	3.49	2.60 ± 2.16	1.95	8.81	<0.001^a^
Angular deviation (degree)	0.58 ± 0.32	0.57	1.36	0.87 ± 0.49	0.75	1.59	0.076^b^

^a^ Linear mixed model comparing the difference between two groups for linear deviation: *p* < 0.05.

^b^ Linear regression comparing the difference between two groups for angular deviation: *p* < 0.05.

For the most part, 3D registration showed less mean deviation than UV match in all four parameters tested: linear trueness, angular trueness, linear precision, and angular precision, and across two devices tested.

## DISCUSSION

This study assessed the accuracy of face scan virtual‐based records using two registration methods: geometry‐based, surface‐based (3D) matching and landmark‐based, and point‐pair (UV) matching. To our knowledge, this is the first study directly comparing these two registration strategies using virtual facebow records. Overall, 3D registration consistently demonstrated superior accuracy compared with UV registration, supporting the use of surface‐based 3D matching during virtual patient superimposition.

The null hypothesis of this study was that there is no difference in accuracy between the 3D match and the UV match. Second, there is no association between devices that acquire facial scans and the registration methods used. The null hypothesis was rejected as statistically significant differences were observed between registration methods, and there is evidence of interaction between device type and registration methods.

These findings are consistent with a systematic review by Marradi et al.,^16^ which reported that 3D registration was the most frequently used method and generally produced lower alignment errors compared to UV or landmark‐based strategies. A key advantage of surface‐based registration is that it leverages rich geometric information across anatomic regions instead of relying on a few sparse points. In addition, the ICP algorithm used in 3D matching minimizes the overall surface‐to‐surface distance across the whole dataset, reducing the likelihood of subtle misalignments that a limited number of landmarks might overlook, consistent with Naudi et al.^15^ Finally, UV registration requires manual landmark identification, which is inherently operator‐dependent and may introduce variability, whereas surface‐based approaches tend to be more objective and reproducible.^16^ Prior studies have similarly reported superior alignment accuracy with 3D matching (approximately 0.12 mm) compared with UV matching, with greater error ranging from 0.16 to 0.47 mm.^10^, ^16^

Recent studies in digital dentistry and image registration further support these concepts. Park et al. demonstrated that automatic 3D registration methods using volumetric CBCT data combined with facial scans can achieve high precision (mean surface alignment error approximately 0.74 mm), highlighting the clinical utility of geometry‐based superimposition.^17^ In contrast, Deztrez et al. evaluated UV registration by mapping 2D photographs into 3D orthodontic models and found that, while UV may be effective for visualization, accuracy may be limited in regions with insufficient distinctive texture.^18^ These findings reinforce the study's finding that 3D registration, which utilizes the complete geometric profile of anatomical structures, is generally more accurate and clinically reliable than UV‐based texture or landmark alignment.

While there is no universally established standard for clinically acceptable deviation in virtual facebow registration within prosthodontics, several studies provide relevant references. In orthognathic surgery, a deviation of less than 2 mm is considered clinically acceptable.^19^ Lin et al. reported mean errors ranging from 0.10 to 0.43 mm for artifact‐resistant surface‐based registration using CBCT and laser‐scanned casts, which they considered clinically acceptable.^8^ Wang et al. suggested 1° and 1 mm deviation as a clinically acceptable error for virtual facebow workflows.^20^ Lam et al. and Lin et al. reported that an angular deviation of less than 1° exhibits good repeatability and is clinically acceptable.^21^, ^22^ Li et al. defined high trueness and precision as approximately 1 mm linear deviation and 1° angulation.^2^ A systematic review on digital surgical guides for implant placement also reported that an acceptable outcome can be achieved with a mean linear deviation of less than 2 mm and an angular deviation of less than 5°.^23^ These findings collectively support that mean linear deviation under 2 mm and angular deviations under 2° are clinically acceptable for many clinical applications.

In this study, 3D registration achieved clinically acceptable deviations (<2 mm), whereas UV registration occasionally exceeded this threshold, particularly for linear precision with the industrial scanner (mean 2.60 mm). The industrial scanner combined with 3D matching demonstrated the highest accuracy for linear trueness (mean 0.98). This excellent repeatability may be attributable to the high fidelity and resolution of industrial scanning and the ability of surface‐based registration to capitalize on dense geometric data.

For angular deviation, 3D matching generated a value between 0.58° and 1.24°, whereas UV matching ranged between 0.87° and 1.88°. Although many values were near or slightly over 1°, angular deviations approaching 2° were observed primarily with UV registration combined with the iPad‐based scanning.

**FIGURE 3:**
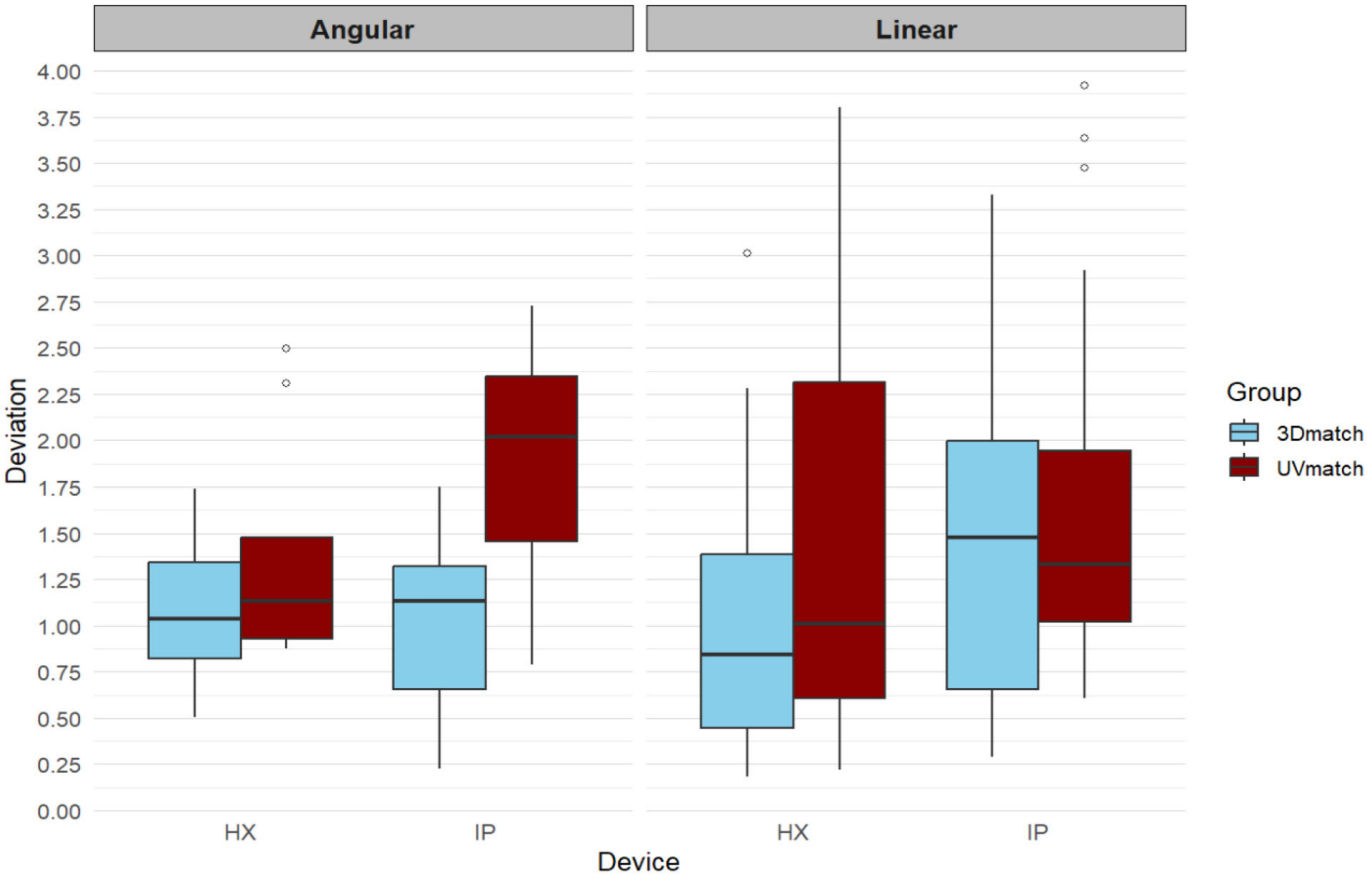
Summary of trueness between two registration methods across devices.

**FIGURE 4:**
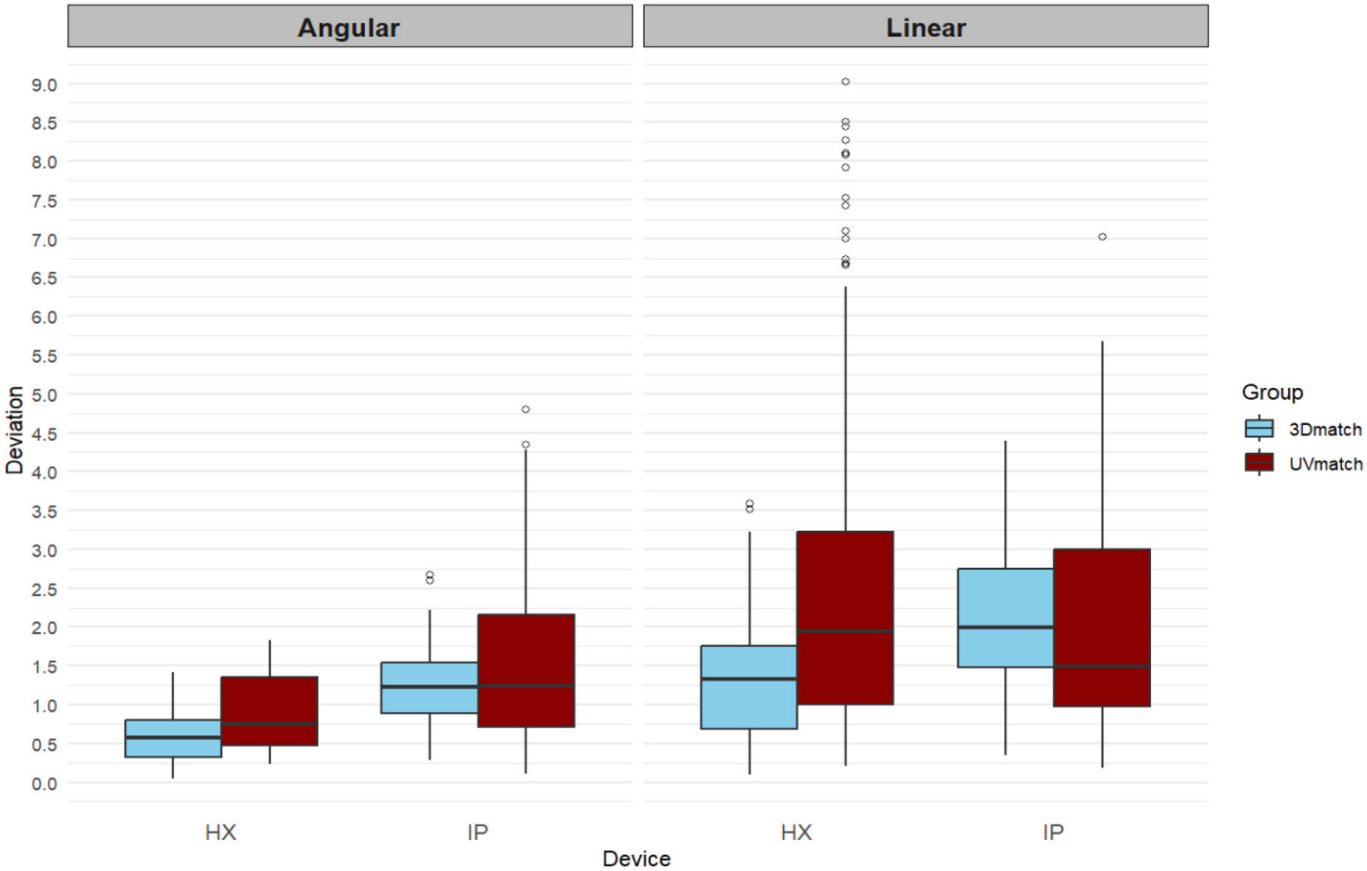
Summary of precision between two registration methods across devices.

Accurate registration is essential in a digital workflow. In this in vitro model, surface‐based 3D registration generally resulted in smaller deviations than landmark‐based UV registration (Figures 3 and 4). A significant interaction was observed between the facial scanning device and virtual facebow transfer accuracy (Table 5). With the iPad scanner, 3D registration significantly improved angular trueness and angular precision, whereas linear outcomes were not significantly different between methods. Conversely, with the industrial scanner, 3D registration significantly improved linear trueness and linear precision, while angular outcomes did not differ significantly. Further studies are warranted to clarify the factors underlying this device–registration interaction and its potential clinical implications.

**TABLE 5:** Summary means comparing between two registration methods and scanners.

	Linear mixed model				
Scanner	Aspects	Accuracy	Mean		*p*‐value
			3D	UV	
iPad	Linear	Trueness	1.48^#^	1.6	0.349
	Linear	Precision	2.09	1.99^#^	0.509
	Angular	Trueness	1.03^#^	1.88	0.002^*^
	Angular	Precision	1.24^#^	1.59	0.025^*^
HX industrial scanner	Linear	Trueness	0.98^#^	1.48	<0.001^*^
	Linear	Precision	1.31^#^	2.6	<0.001^*^
	Angular	Trueness	1.06^#^	1.36	0.248
	Angular	Precision	0.58^#^	0.87	0.076^*^

# When comparing the mean deviation both for linear as well asangular deviation, mean deviation of 3D in general is lower than mean deviationof UV registration.

* There is evidence of a unique interaction between registration method and the type of facial scanning device.When using the iPad‐based scanner, 3D registration yielded significantly higheraccuracy than UV match registration for angular parameters. In contrast, withthe industrial‐grade scanner, 3D registration provided superior accuracy than UV match for linear parameters *p*‐value <0.05).

Overall, the result of this study underscores the importance of selecting an appropriate registration strategy in the digital workflow. Surface‐based 3D registration demonstrated consistently superior performance across both devices, supporting its use as a clinically reliable approach for virtual facebow transfer. The integration of 3D surface‐based registration with high‐resolution scanners represents the highest accuracy of virtual facebow transfer.

This study acknowledges several limitations. Only one type of industrial scanner and one brand of iPad with a single software were used. Consequently, the outcome is confined to the device and software used. Second, as this study employed a manikin model that lacks the elasticity and soft‐tissue characteristics of the human face, the findings may not entirely reflect clinical reality. Validation through studies involving real patient samples is warranted.

Third, the facebow fork was additively manufactured (Form 3+; Formlabs) using a photopolymer resin. Additive manufacturing can introduce dimensional deviations influenced by build orientation, support strategy, and post‐processing, which may affect registration by altering fork seating repeatability and the geometry available for superimposition.^24^ Because the same fork and manufacturing workflow were used across all groups, any printing‐related deviation is best interpreted as a potential generic error affecting absolute values rather than the between‐method comparisons within this standardized setup. Different anatomical facebow fork designs are expected to influence registration accuracy because rigidity, repeatable stabilization, and the amount or distribution of distinctive geometry can affect the robustness of scanning and alignment.^25^ As this study consistently uses a single facebow fork design, it does not affect the accuracy of the study. However, the outcome of this study is limited to the design of our facebow fork. Future studies should compare multiple facebow fork designs and accuracy of the virtual facebow.

## CONCLUSIONS

This study demonstrated that 3D match registration generally achieved greater accuracy than UV match registration. Importantly, there is evidence of a unique interaction between registration method and the type of facial scanning device. When using the iPad‐based scanner, 3D registration yielded significantly higher accuracy than UV match registration for angular parameters. In contrast, with the industrial‐grade scanner, 3D registration provided superior accuracy than UV match for linear parameters. In this study, the industrial scanner consistently produced lower mean error values across all measured parameters compared to the iPad scanner, indicating its superior overall performance. These findings suggest that the combination of 3D registration with an industrial scanner offers the highest level of accuracy and reliability for virtual maxillary arch transfer.

## CONFLICT OF INTEREST STATEMENT

The authors declare no conflicts of interest.

## Data Availability

The data that support the findings of this study are available from the corresponding author upon reasonable request.
